# Dendritic Cells Transfected with MHC Antigenic Determinants of CBA Mice Induce Antigen-Specific Tolerance in C57Bl/6 Mice

**DOI:** 10.1155/2020/9686143

**Published:** 2020-09-04

**Authors:** Sergey V. Sennikov, Valeriy P. Tereshchenko, Vasiliy V. Kurilin, Julia A. Shevchenko, Julia A. Lopatnikova, Alexander N. Silkov, Amir Z. Maksyutov, Maria S. Kuznetsova, Nadezda Y. Knauer, Aleksey S. Bulygin, Julia N. Khantakova

**Affiliations:** ^1^Federal State Budgetary Scientific Institution “Research Institute of Fundamental and Clinical Immunology” (RIFCI), 630099 Novosibirsk, Russia; ^2^Novosibirsk State University, 630090 Novosibirsk, Russia; ^3^State Research Center of Virology and Biotechnology “Vector”, 630559 Koltsovo, Russia

## Abstract

**Background:**

Nonspecific immunosuppressive therapy for graft rejection and graft-versus-host disease (GVHD) is often accompanied by severe side effects such as opportunistic infections and cancers. Several approaches have been developed to suppress transplantation reactions using tolerogenic cells, including induction of FoxP3^+^ Tregs with antigen-loaded dendritic cells (DCs) and induction of CD4^+^IL-10^+^ cells with interleukin IL-10-producing DCs. Here, we assessed the effectiveness of both approaches in the suppression of graft rejection and GVHD.

**Methods:**

IL-10-producing DCs were generated by the transfection of DCs with DNA constructs encoding mouse IL-10. Antigen-loaded DCs from C57BL/6 mice were generated by transfection with DNA constructs encoding antigenic determinants from the H2 locus of CBA mice which differ from the homologous antigenic determinants of C57BL/6 mice.

**Results:**

We found that both IL-10-producing DCs and antigen-loaded immature DCs could suppress graft rejection and GVHD but through distinct nonspecific and antigen-specific mechanisms, respectively. *Discussion*. We provide data that the novel approach for DCs antigen loading using DNA constructs encoding distinct homologous determinants derived from major histocompatibility complex genes is effective in antigen-specific suppression of transplantation reactions. Such an approach eliminates the necessity of donor material use and may be useful in immunosuppressive therapy side effects prevention.

## 1. Introduction

Currently, transplantation is the therapy of choice during the late stages of organ damage or failure. Although nonspecific immunosuppressive therapy significantly increases the chance of successful allograft survival, it is often accompanied by severe side effects such as opportunistic infections and cancers [1]. In addition, immunosuppressive therapy does not prevent the development of chronic rejection reactions [2]. Therefore, biological approaches to induce antigen-specific immunological tolerance to transplanted organs and tissues are promising avenues.

Several regulatory cell subpopulations are involved in the development and maintenance of immunological tolerance. Regulatory cells, such as FoxP3^+^ Tregs, CD4^+^IL-10^+^ cells, and tolerogenic dendritic cells (tol-DCs), function through similar immune mechanisms and interact with one another. These cell types are involved in the induction of peripheral tolerance to self-antigens and limit immune responses to foreign antigens [3]. It also can be used for suppression of immune reactions to transplanted alloantigens.

It was previously reported that interleukin IL-10-producing DCs induce CD4^+^IL-10^+^ cells *in vitro* and *in vivo* [4, 5], and CD4^+^IL-10^+^ cells, in turn, induce T cell anergy *in vitro* and impede graft-versus-host disease (GVHD) *in vivo* [5, 6]. Tolerogenic allo-DCs or tolerogenic DCs loaded with specific antigens induce functional FoxP3^+^ Tregs [7, 8]. Such DCs induce T cell unresponsiveness in culture and prolong allograft survival [9, 10]. Tregs generated by antigen-loaded DCs were shown to prevent GVHD [7].

Transplantation reactions such as graft rejection reaction and GVHD are known to develop when the major histocompatibility complex (MHC) antigens of the donor and the recipient are not a perfect match. Despite improvements in genotyping and selection of donor-recipient pairs, the complete antigenic similarity is often not achieved [11]. In this context, it seems quite reasonable to investigate the approach involving alloantigen-specific immune mechanisms to suppress transplantation reactions. The use of host immature dendritic cells pulsed with allogeneic MHC peptide, while combined with systemic immunosuppressive therapy was shown to be sufficient to suppress graft rejection, thus providing long-term allograft acceptance [12].

Besides, one more promising approach is known where IL-10-producing DCs are capable of local suppression of immune reactions [5] that allows its use instead of systemic immunosuppressive therapy.

Thus, within the present study, we decided to investigate three approaches to reduce alloreactivity to transplanted tissues: the use of (i) IL-10-producing DCs, (ii) antigen-loaded immature DCs, and (iii) antigen-loaded IL-10-producing DCs, respectively. IL-10-producing DCs were generated by the transfection of DCs with DNA constructs encoding mouse IL-10. To generate antigen-loaded DCs, we transfected immature DCs from C57Bl/6 mice with DNA constructs encoding antigenic determinants from the H2 locus of CBA mice which differ from the homologous antigenic determinants of C57BL/6 mice. The aim of such approach was to develop a novel strategy for antigen-specific transplantation reactions suppression which may be more relevant for future clinical applications. To generate antigen-loaded IL-10-producing DCs, we used simultaneous transfection with both DNA constructs. We assessed the effectiveness of generated DC types in alloreactivity suppression in mixed lymphocyte culture (MLC) assays *in vitro* as well as in acute GVHD and skin graft transplantation models *in vivo*.

## 2. Materials and Methods

### 2.1. Ethics Statement

All experimental protocols and procedures were approved by the institutional review board of the Research Institute of Fundamental and Clinical Immunology, Novosibirsk, Russian Federation (Protocol No. 99/2016-02-09). The study followed the principles outlined in the Declaration of Helsinki for all human and animal experimental investigations.

### 2.2. Animals

Female C57BL/6, CBA, CBF1 (F1: C57BL/6 × CBA), and BALB/c mice were obtained from a breeding farm of the Institute of Cytology and Genetics (Novosibirsk, Russia). The mice were maintained in the animal facility of the Research Institute of Fundamental and Clinical Immunology and fed a standard diet under natural light conditions with unrestricted access to food and water. Mice aged 2–6 months were used in the study.

### 2.3. Plasmid DNA Constructs

**Figure 1:**
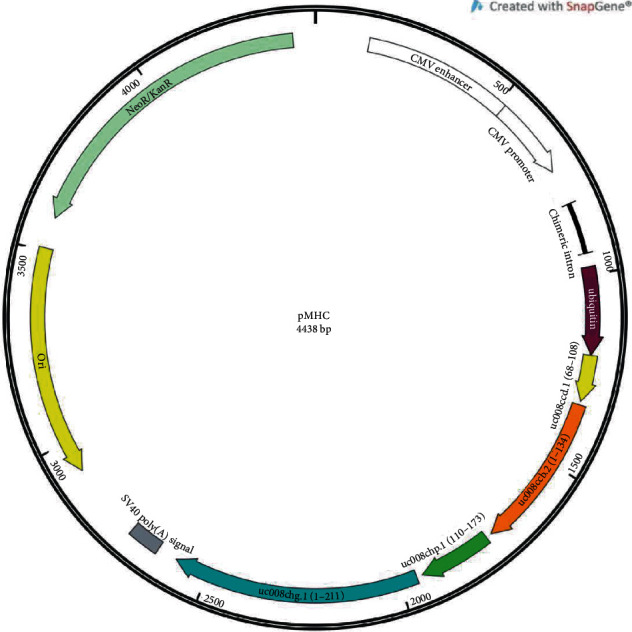
pMHC plasmid map.

The following plasmid DNA constructs were used in the study: (i) pIL-10, a pmax-pIL-10 construct encoding mouse IL-10; (ii) pMHC, a pmax-MHC construct encoding antigenic determinants from the H2 locus of CBA mice (H2k allele), which differ from the homologous antigenic determinants of C57BL/6 mice (H2b allele); the pMHC diagram map is shown in Figure 1, and the full amino acid sequence encoded by pMHC is shown in Supplementary material as Suppl. Fig. S1; and (iii) p5, a control construct encoding antigenic determinants from the H2 locus of CBA mice, which do not differ from the homologous antigenic determinants of C57BL/6 mice. The generation of plasmid DNA constructs is described in the results section.

### 2.4. Generation of Transfected DCs and Tolerogenic Cultures

Immature DCs were generated using a C57BL/6 mouse bone marrow monocyte pool. Briefly, adherent bone marrow cells were cultured in 25 cm^2^ flasks (TPP, Switzerland) at a density of 1 × 10^6^/mL in RPMI-1640 (Biolot, Russia) supplemented with 10% FCS (Biowest, France), 2 mM L-glutamine (Biolot, Russia), 10 mM HEPES (Biolot, Russia), 5 × 10^−4^ M 2-mercaptoethanol (Sigma-Aldrich, USA), 80 *μ*g/mL gentamycin (KRKA, Slovenia), 100 *μ*g/mL benzylpenicillin (Biolot, Russia), 20 ng/mL rmGM-CSF (R&D Biosystems, USA) and 20 ng/mL rmIL-4 (R&D Biosystems, USA). Half of the culture medium and growth factors were refreshed every second day. After 3 days of culture, 65.15 ± 5.21% (mean ± SD) of cells in cultures were CD11c^+^H2-b^+^ DCs. The viability of obtained DCs according to DAPI incorporation was 99.34 ± 0.38% (mean ± SD).

Transfection of DCs was carried out by electroporation after 3 days of culture. Briefly, 2.0 × 10^7^/mL of DCs in 100 *μ*L of OptiMem (Thermo Fisher Scientific, USA) were electroporated using a BTX 830 electroporator (BTX, USA) with a pulse strength of 260 V, a pulse duration of 5 ms, and a plasmid DNA concentration of 60 *μ*g/mL. For simultaneous electroporation of DCs with pIL-10 and pMHC, 30 *μ*g/mL of each plasmid was used. DCs transfected with the p5 plasmid were used as controls.

To assess the phenotype of transfected DCs, cells were cultured after electroporation in a 48-well plate at a density of 1 × 10^6^/mL without growth factors for 1 day.

To produce tolerogenic cultures, electroporated DCs were cocultured with autologous splenocytes at ratio 1 : 10 in culture medium for 4 days. The tolerogenic potential of the obtained cultures was assessed based on the frequencies of FoxP3^+^ Tregs and IL-10^+^CD4^+^ cells as well as on suppression of proliferation in response to allostimulation in mixed lymphocyte cultures, skin grafting, and GVHD models.

### 2.5. Flow Cytometry

For flow cytometry, 2 × 10^5^ cells were collected and incubated in the dark at room temperature with an appropriate combination of fluorescent monoclonal antibodies in staining buffer for 20 min. DC phenotype was assessed using anti-CD11c-FITC (N418), anti-H-2D^b^-PE (KH95), anti-CD83-PE-Cy7 (Michel-19), anti-CD86-APC-Cy7 (GL-1), anti-CD80-Bv421 (16-10A1), and anti-CD40-PE-Cy5 (3/23) antibodies (BioLegend, USA). For FoxP3^+^ Treg and CD4^+^IL-10^+^ cell staining, anti-CD4-PerCP(RM4-5), anti-CD25-PE(PC61), anti-IL-10-PE(JES5-16E3), and anti-FoxP3-APC(FJK-16s), and antibodies (BioLegend, USA) were used. After incubation with surface antibodies, the cells were washed in 500 *μ*L of phosphate-buffered saline (PBS). DC gating strategy and representative histograms of DCs phenotyping are shown in Supplementary Figure S2.

For intracellular analysis after staining with surface antibodies, cells were fixed in PBS solution containing 1% paraformaldehyde, permeabilized with a 0.1% (*v*/*v*) Tween-20 solution, and stained with anti-IL-10-PE (JES5-16E3) or anti-FoxP3-APC (FJK-16s) monoclonal antibodies. Gating strategies for CD4^+^CD25^+^FoxP3^+^ Tregs and CD4^+^IL-10^+^ cells and examples of individual samples are shown in Supplementary Figure S3.

### 2.6. Mixed Lymphocyte Culture (MLC) Assay

The proliferation of tolerogenic cultures in response to allostimulation was evaluated in an MLC assay. C57Bl/6 tolerogenic cultures containing transfected DCs and autosplenocytes (1 : 10) were cocultured at a 1 : 1 ratio with mitomycin C-treated allosplenocytes of CBA or BALB/c mice (Tol.Culture^+^AlloSpl-MytC group). CBA or BALB/c splenocytes were treated with 50 *μ*g/mL of mitomycin C in PBS for 30 minutes followed by 3 washes in medium containing 10% FCS. Tolerogenic cultures without allogeneic splenocytes stimulation (Tol.Culture group) were used as controls. After 3 days, the degree of proliferation was evaluated by measuring the optical density (OD) using a PreMix WST-1 kit (Takara, Japan) according to the manufacturer's instructions. The proliferation suppression index in the MLC was calculated using the following formula: OD [Tol.Culture]/OD [Tol.Culture^+^AlloSpl − MytC].

### 2.7. Acute GVHD Model

Acute GVHD was induced by intravenous injection of hybrid CBF1 (C57BL/6 × CBA) or BALB/c mice with C57BL/6 tolerogenic cultures. Mice injected with saline and splenocytes cultured without DCs were used as controls. Each mouse was intravenously administered a single dose of 1 × 10^8^ cells in 0.5 mL of saline. GVHD activity was assessed based on changes in spleen weight 2 and 3 weeks after the administration of tolerogenic cultures.

### 2.8. Skin Graft Model

Skin grafts from CBA or BALB/c mice were transplanted to С57BL/6 mice. C57BL/6 mice transplanted with syngeneic skin grafts were used as engraftment controls. The day before skin graft transplantation, tolerogenic cultures (1 × 10^6^ cells in 100 *μ*L of saline) were subcutaneously injected in the withers of C57Bl/6 mice. Mice injected with 100 *μ*L of saline, syngeneic splenocytes, and splenocytes stimulated with nontransfected DCs in a similar volume as the experimental groups were used as controls. A skin graft (0.25 cm^2^) was prepared from the tail skin of CBA or BALB/c mice on the day of transplantation. An operation bed for skin grafts was formed in the backs of C57BL/6 recipient mice. The mice were anesthetized using 2% isoflurane. After transplantation, the skin graft was fixed with a tight bandage. Mice from different experimental groups were placed in separate cages with unrestricted access to food and water. Readministration of appropriate cellular preparations or saline was performed on day 3 after skin graft transplantation.

Transplantation of a syngeneic skin graft was successful and not accompanied by postoperative complications in 90–95% of mice. The skin grafts of transplanted mice were monitored starting at day 7 postsurgery. The rejection was assessed *via* necrotic changes in the transplanted skin graft. Grafts with >80% necrosis were considered rejected. Skin grafts that became necrotic by day 7 postsurgery were considered transplantation technical errors and were not included in the analysis.

### 2.9. Statistical Analysis

The normality of data was assessed using the Shapiro-Wilk normality test. Normally distributed data were analyzed using parametric one-way ANOVA with Tukey's corrected multiple comparisons. Values of *P* ≤ 0.05 were considered statistically significant. The normally distributed data (mean ± SD) are indicated in the figures. Abnormally distributed data were analyzed using the nonparametric Kruskal-Wallis test with Dunn's corrected multiple comparisons. Values of *P* ≤ 0.05 were considered statistically significant. For abnormally distributed data, the median and interquartile range are indicated in figures. Survival curves were compared using the Mantel-Cox test and log-rank test for trend. Values of *P* ≤ 0.05 were considered statistically significant. Statistical analysis was performed using Prism 7.0 (GraphPad Software, La Jolla, CA, USA).

## 3. Results

### 3.1. Generation of Plasmid DNA Constructs

To design pMHC DNA plasmids encoding MHC antigenic determinants, we performed bioinformatic analyses of the genomes of two mouse strains (C57BL/6 and CBA). Mouse genotypes were obtained from the Mouse Genomes Project website (https://www.sanger.ac.uk/science/data/mouse-genomes-project). All genes whose names contained the word “histocompatibility” and were present in the H2 locus of chromosome 17 were preliminarily classified as histocompatibility genes. Fragments of these genes with noticeable polymorphisms between mouse strains (uc008ccd.1 (68-108), uc008ccb.2 (1-134), uc008chp.1 (110-173), and uc008chg.1 (1-211)) were selected for inclusion in the DNA construct (Supplementary Figure S1). According to *in silico* analysis performed on the http://www.iedb.org/ website, the included fragments contained T cell epitopes from the CBA H2 locus (H2-k allele), which differed from epitopes in the C57BL/6 H2 locus (H2-b allele). To improve processing, DNA encoding a modified ubiquitin with a substituted C-terminal amino acid {G76V} (UbV76) was added to the N-terminus of the construct [13, 14]. Sequences of fragments were connected *via* a GPGPG linker. The design of recombinant genes was carried out using Gene Designer 2.0 (DNA2.0, Inc.). Cloning of recombinant genes into the pmax-Ub vector and production of DNA constructs encoding target proteins were performed as previously described [13, 14].

In p5 control plasmids, fragments of genes lacking noticeable polymorphisms were included. For pIL-10 plasmids, the amino acid sequence of mouse IL-10 was obtained from the UniProtKB database (P18893).

### 3.2. Phenotype of Transfected DCs and Induction of Regulatory T Cells

DCs were generated from the bone marrow of C57BL/6 mice following cultivation for 3 days with rmGM-CSF and rmIL-4. The resulting DCs were transfected with plasmid DNA constructs by electroporation. The efficiency of pMHC transfection was considered comparable with pmax-GFP plasmid (encoding green fluorescent protein) transfection efficiency since both plasmids were designed using pmax vector and have comparable sizes (4.44 kbp and 3.617 kbp, respectively). pGFP transfection efficiency of viable cells was 32.17 ± 4.48% (mean ± SD) as shown in our previous work [15]. According to DAPI incorporation, cell death after pMHC transfection was 7.3 ± 2.93% (mean ± SD). An optimization of pIL-10 transfection parameters was as well performed previously [15]. We used ELISA for soluble IL-10 quantification in the culture medium of cells transfected with different amounts of the pIL-10 [15].

**Figure 2:**
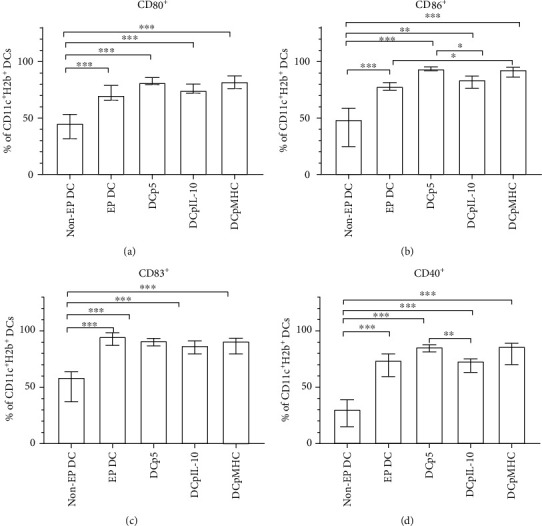
Relative quantities of cells expressing surface markers (a) CD80^+^, (b) CD86^+^, (c) CD83^+^, and (d) CD40^+^ among groups of electroporated and nonelectroporated DCs. *N* = 12 per group. Data are presented as median and interquartile range. Brackets indicate significant differences (Kruskal-Wallis test with Dunn's corrected multiple comparisons, ∗*P* < 0.05, ∗∗*P* < 0.01, ∗∗∗*P* < 0.001). Non-EP DC: nonelectroporated DCs; EP DC: DCs electroporated without a plasmid; DCp5: DCs electroporated with a control plasmid encoding antigenic determinants from the H2 locus of CBA mice, which do not differ from the homologous antigenic determinants of C57BL/6 mice; DCpIL-10: DCs electroporated with a plasmid encoding mouse IL-10; DCpMHC: DCs electroporated with a plasmid encoding antigenic determinants from the H2 locus of CBA mice, which differ from the homologous antigenic determinants of C57BL/6 mice.

First, we assessed the phenotype of transfected DCs, as immature DCs are known to express relatively lower levels of costimulatory and activation molecules and can thus exert tolerogenic function [10]. We found that all electroporated DCs demonstrated more mature phenotype according to surface expression of CD80, CD86, CD40, and CD83 compared with nonelectroporated cells (Figure 2). This effect is known to be associated with electroporation itself that triggers the maturation of DCs [16]. Electroporation with pIL-10 slightly decreased the expression levels of CD86 and CD40. DC gating strategy and representative histograms of DCs phenotyping are shown in Supplementary Figure S2.

**Figure 3:**
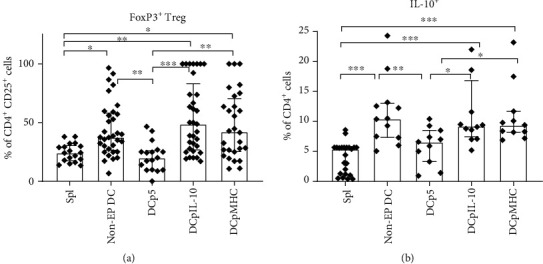
Tolerogenic properties of the obtained DCs. Induction of (a) CD4^+^CD25^+^FoxP3^+^ Tregs and (b) IL-10-producing CD4^+^ T cells by the different DC groups in C57BL/6 mouse splenocyte cultures. (a) *N* = 23 per group, (b) *N* = 10 per group. Data are presented as median and interquartile range. Brackets indicate significant differences (the Kruskal-Wallis test with Dunn's corrected multiple comparisons, ∗*P* < 0.05, ∗∗*P* < 0.01, and ∗∗∗*P* < 0.001). Spl: C57Bl/6 splenocytes cultured without adding DCs; non-EP DCs: C57Bl/6 splenocytes cocultured with nonelectroporated DCs; DCp5: C57Bl/6 splenocytes cocultured with DCs electroporated with p5; DCpIL-10: C57Bl/6 splenocytes cocultured with DCs electroporated with pIL-10; DCpMHC: C57Bl/6 splenocytes cocultured with DCs electroporated with pMHC.

It is currently well known that immature DCs play an important tolerogenic role *in vivo*, taking part in anti-inflammatory processes [17, 18]. During the GVHD or graft rejection processes, however, a proinflammatory molecular context takes place, resulting in DC maturation. In our study, we aimed to obtain DCs with tolerogenic properties *via* transfection with pMHC and pIL-10 DNA constructs. But the transfected DCs showed some shift towards a more mature phenotype, so it was important to check the possible negative effect on their tolerogenic function. To this end, we cocultured the transfected DCs with autologous splenocytes for 4 days and assessed the frequency of FoxP3^+^ Tregs and IL-10^+^CD4^+^ cells in the cultures. Gating strategies for CD4^+^CD25^+^FoxP3^+^ Tregs and CD4^+^IL-10^+^ cells and examples of individual samples are shown in Supplementary Figure S3. The DCs transfected with pIL-10 as well as pMHC-transfected DCs were shown to induce both FoxP3^+^ Tregs and IL-10^+^CD4^+^ cells at similar levels to nonelectroporated immature DCs, which, in turn, was expectedly heightened due to the immaturity of the cells (Figure 3). The frequencies of FoxP3^+^ Tregs and IL-10^+^CD4^+^ cells were higher in splenocytes stimulated with nonelectroporated, pIL-10-electroporated, and pMHC-electroporated DCs compared with splenocytes stimulated with DCs transfected with the control plasmid p5 or nonstimulated splenocytes. Thus, the DCs electroporated with pIL-10 and pMHC had shown the suppression potential despite their more mature phenotype.

### 3.3. *In Vitro* Suppression of Allospecific Responses by Transfected DCs

Modulation of allospecific immune responses by transfected DCs was assessed using the MLC assay. Mitomycin C-treated splenocytes from CBA or BALB/c mice were added to tolerogenic cultures containing the C57Bl/6 transfected DCs and autologous splenocytes (1 : 10) as stimulators. The proliferation suppression index in each MLC was calculated using the formula: [proliferation of tolerogenic culture without stimulation]/[proliferation of tolerogenic culture stimulated by allosplenocytes].

**Figure 4:**
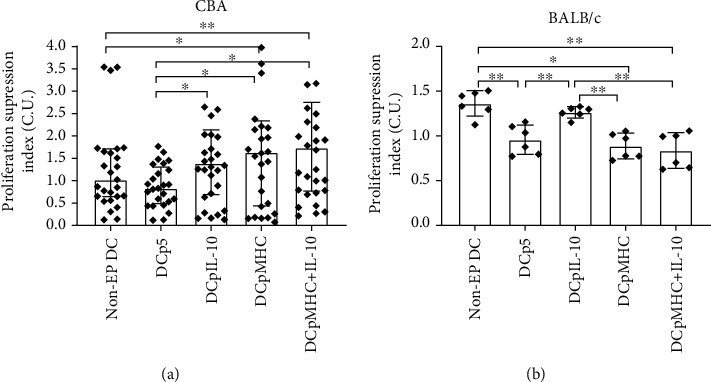
Suppression of proliferation of C57BL/6 mouse splenocytes cocultured with transfected DCs through adding (a) CBA mouse splenocytes (*N* = 22 per group) or (b) BALB/c mouse splenocytes (*N* = 6 per group). Brackets indicate significant differences. (a) The data are presented as median and interquartile range (the Kruskal-Wallis test with Dunn's corrected multiple comparisons, *P* ≤ 0.05). (b) The data are presented as mean and SD (one-way ANOVA with Tukey's corrected multiple comparisons, ∗*P* < 0.05, ∗∗*P* < 0.01). Non-EP DCs: C57Bl/6 splenocytes cocultured with nonelectroporated DCs stimulated with CBA or BALB/c splenocytes; DCp5: C57Bl/6 splenocytes cocultured with DCs electroporated with p5 stimulated with CBA or BALB/c splenocytes; DCpIL-10: C57Bl/6 splenocytes cocultured with DCs electroporated with pIL-10 stimulated with CBA or BALB/c splenocytes; DCpMHC: C57Bl/6 splenocytes cocultured with DCs electroporated with pMHC stimulated with CBA or BALB/c splenocytes; DCpMHC^+^IL-10: C57Bl/6 splenocytes cocultured with DCs electroporated with pMHC and pIL-10 stimulated with CBA (a) or BALB/c (b) splenocytes.

In MLC assay, the DCs transfected, respectively, with pIL-10, pMHC, or simultaneously with pIL-10 and pMHC had more pronounced abilities to suppress proliferation of autologous splenocytes in response to stimulation with CBA splenocytes, as compared with the control DCs (Figure 4(a)).

Since the use of the DCs loaded with H2 antigenic determinants of CBA mice (the cells electroporated with pMHC) aimed at inducing the antigen-specific suppression of immune reactions, the specificity of the suppression induced was assessed using MLCs with BALB/c splenocytes as stimulators. In response to stimulation with BALB/c splenocytes, the pIL-10-transfected DCs, but not the pMHC-transfected DCs, maintained their ability to suppress proliferation of autologous splenocytes (Figure 4).

The ability to suppress proliferation of autologous splenocytes was detected also for the nonelectroporated DCs in MLCs using BALB/c splenocytes, but not in MLCs using CBA splenocytes. These observations are also consistent with the fact that immature DCs, what the non-EP DCs are, do have tolerogenic potential. [17, 18]. Besides, the BALB/c's MHCs are more antigenic to C57Bl/6 mice than the CBA's MHCs, as in MLC, the BALB/c–C57Bl/6 responses are rather detected than CBA–C57Bl/6 responses [19, 20].

In summary, MLC assays showed that the DCs transfected with pIL-10 suppressed the proliferation of autologous splenocytes in an antigen-independent manner. In contrast, DCs transfected by with H2 antigenic determinants of CBA mice suppressed proliferation in response to CBA antigens but not to BALB/c antigens.

### 3.4. Suppression of Acute GVHD by Transfected DCs

**Figure 5:**
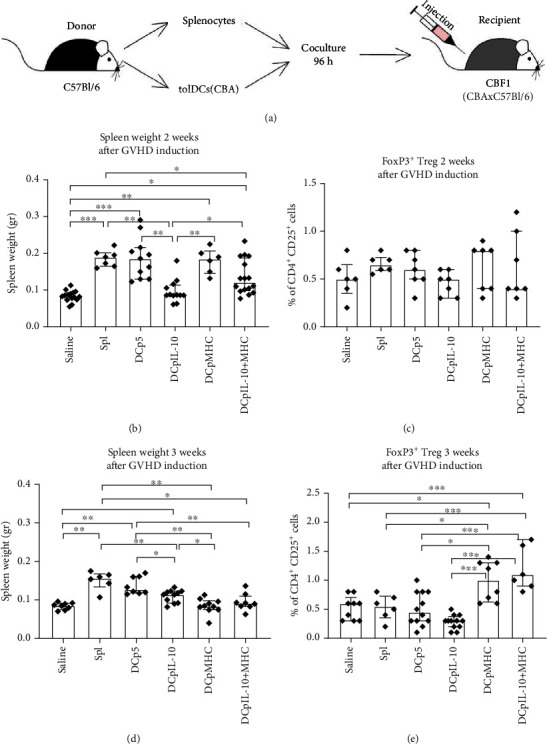
Induction of GVHD in CBF1 (F1: C57BL/6×CBA) mice by splenocytes cocultured with the transfected DCs. (a) A GVHD induction scheme. Spleen weights were measured (b) 2 weeks or (d) 3 weeks after induction of GVHD (*N* = 10 per group; the Kruskal-Wallis test with Dunn's corrected multiple comparisons, *P* ≤ 0.05). Frequencies of FoxP3^+^ Tregs were measured in mouse spleens (c) 2 weeks or (e) 3 weeks after induction of GVHD (*N* = 7 per group; one-way ANOVA with Tukey's corrected multiple comparisons, *P* ≤ 0.05). Median and interquartile range. Brackets indicate significant differences, ∗*P* < 0.05, ∗∗*P* < 0.01, ∗∗∗*P* < 0.001. Saline: administration of saline; Spl: administration of C57BL/6 mouse splenocytes; DCp5: administration of cocultures of autosplenocytes and DCs electroporated with a control plasmid p5; DCpIL-10: administration of cocultures of autosplenocytes and DCs electroporated with a pIL-10; DCpMHC: administration of cocultures of autosplenocytes and DCs electroporated with a pMHC; DCpMHC^+^IL-10: administration of cocultures of autosplenocytes and DCs electroporated with pMHC and pIL-10 simultaneously.

Acute GVHD was induced *in vivo* by intravenous injection of C57Bl/6 splenocytes or tolerogenic cultures containing C57Bl/6 transfected DCs and autologous splenocytes into CBF1 (F1: CBA×C57BL/6) mice (Figure 5(a)). GVHD induced by splenocytes alone was accompanied by splenomegaly, expansion of CD3^+^ and CD8^+^, and reduction of CD19^+^ and CD4^+^ spleen lymphocytes (Supplementary Figure S4). Therefore, the ability of transfected DCs to suppress GVHD was assessed using the detection of spleen weight reduction.

Two weeks following the induction of acute GVHD, we observed a twofold increase in the spleen weights of the mice receiving C57BL/6 splenocyte monocultures (a group of GVHD induction) and cultures containing the p5- and pMHC-transfected DCs compared with the non-GVHD mice (administration of saline) (Figure 5(b)). Mice administered the DCpIL-10 tolerogenic cultures had normal spleen weights. The median spleen weights of mice receiving tolerogenic cultures containing the DCs simultaneously transfected with pIL-10 and pMHC were intermediate between those of mice with and without induction of GVHD. Two weeks following the GVHD induction, there were no significant differences in FoxP3^+^ Treg frequency in the host spleens of any groups (Figure 5(c)).

Three weeks after induction of GVHD, we found that higher spleen weights were preserved in mice with acute GVHD (administration of splenocytes) as well as in the control group receiving DCp5 tolerogenic cultures (Figure 5(d)). At this time, the median spleen weight of mice receiving tolerogenic cultures containing DCpIL-10 was significantly higher than the median spleen weight of mice without the GVHD induction (administration of saline) but significantly lower than the median spleen weight of mice with the GVHD induction (administration of splenocyte monocultures). The spleen weights of mice administered tolerogenic cultures containing the DCs transfected with pMHC and pIL-10^+^pMHC did not differ significantly from those of mice without the GVHD induction and were significantly lower than then spleen weights of mice with GVHD. In agreement with this finding, after 3 weeks, the frequencies of FoxP3^+^ Treg cells in the spleens of host CBF1 mice were increased 2.5-fold when tolerogenic cultures contained the DCs transfected with pMHC alone or in combination with pIL-10 (Figure 5(e)) were injected.

**Figure 6:**
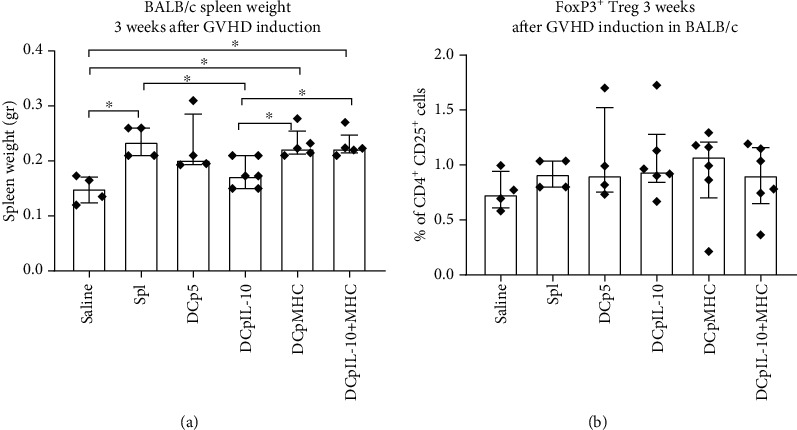
Induction of GVHD in BALB/c mice by splenocytes cocultured with transfected DCs. (a) Spleen weight of BALB/c host mice 3 weeks after GVHD induction. (b) FoxP3^+^ Treg frequencies in host mouse spleens 3 weeks after GVHD induction. *N* = 4 per group. Median and interquartile range. Brackets indicate significant differences (the Kruskal-Wallis test with Dunn's corrected multiple comparisons, ∗*P* < 0.05). Saline: administration of saline; Spl: administration of C57BL/6 mouse splenocytes; DCp5: administration of cocultures of autosplenocytes and DCs electroporated with a control plasmid p5; DCpIL-10: administration of cocultures of autosplenocytes and DCs electroporated with a pIL-10; DCpMHC: administration of cocultures of autosplenocytes and DCs electroporated with a pMHC; DCpIL-10^+^MHC: administration of cocultures of autosplenocytes and DCs electroporated with pMHC and pIL-10 simultaneously.

To confirm the antigen-specific suppression by DCpMHC, we assessed the induction of GVHD in BALB/c mice. We found that the spleens of host BALB/c mice in the DCpMHC and DCpIL-10^+^MHC groups were enlarged 3 weeks following the GVHD induction (Figure 6(a)), unlike the spleens of host CBF1 mice at that time period. In the case of GVHD in BALB/c mice, the graft rejection reaction was not blocked and may have affected the result. However, BALB/c mice administered DCpIL-10 tolerogenic cultures had lower spleen weights compared with mice with GVHD (administration of splenocyte monocultures). In addition, 3 weeks following the GVHD induction, we did not observe higher FoxP3^+^ Treg frequencies in BALB/c spleens upon administration of tolerogenic cultures containing DCpMHC and DCpIL-10^+^pMHC, unlike CBF1 mice (Figure 6(b)).

### 3.5. Suppression of Skin Graft Rejection by Transfected DCs

**Figure 7:**
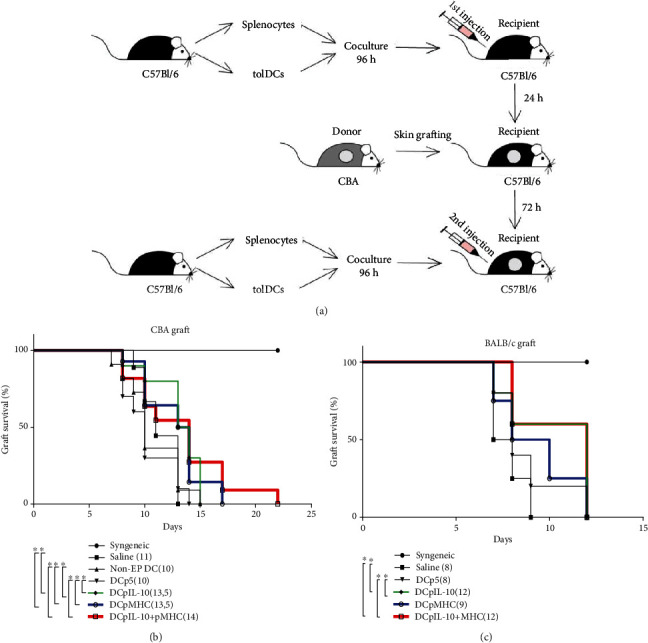
Suppression of allogeneic skin graft rejection by splenocytes cocultured with transfected DCs. Experimental design (a) and survival curves for (b) CBA and (c) BALB/c skin grafts. (b) *N* = 10. (c) *N* = 4. The median graft survival time has indicated in round brackets for each group, where applicable. Square brackets indicate significant differences (the Mantel-Cox test and log-rank test for trend, ∗*P* < 0.05). Syngeneic: transplantation of syngeneic skin graft without other interventions (positive control); Saline: administration of saline; non-EP DC: administration of nonelectroporated DCs; DCp5: administration of cocultures of autosplenocytes and DCs electroporated with a control plasmid p5; DCpIL-10; administration of cocultures of autosplenocytes and DCs electroporated with pIL-10; DCpMHC: administration of cocultures of autosplenocytes and DCs electroporated with pMHC; DCpIL-10^+^MHC; administration of cocultures of autosplenocytes and DCs electroporated with pMHC and pIL-10 simultaneously.

To assess the ability of the transfected DCs to suppress solid organs rejection, we transplanted skin grafts from CBA or BALB/c mice (donors) to C57BL/6 mice (recipients) (Figure 7(a)). C57BL/6 tolerogenic cultures containing transfected DCs (1 × 10^6^ cells) and autologous splenocytes (1 : 10) were subcutaneously administered to withers of syngeneic animals twice: 24 h prior to transplantation and 72 h posttransplantation. Mice receiving syngeneic transplants and mice injected with saline upon allogeneic transplantation served as positive and negative controls, respectively. Rejection of skin grafts was assessed every day starting on day 7.

The syngeneic skin grafts were not rejected. The median survival time for allogeneic grafts upon saline administration was 11 days for the CBA skin grafts and 8 days for the BALB/c skin grafts (negative controls) (Figures 7(b) and 7(c)). When CBA mice were as donors, the median graft survival time in the mice administered nonelectroporated DCs or DCp5-pretreated splenocytes had no significant differences from that of negative controls (10 and 10 days, respectively). The use of the DCpIL-10, the DCpMHC, and the DCpIL-10^+^MHC tolerogenic cultures significantly extended the median survival time of skin grafts compared to control groups (13.5, 13.5, and 14 days, respectively) (Figure 7(b)).

In BALB/c skin graft experiments, the median survival time of skin graft in mice administered the DCp5 and the DCpMHC tolerogenic cultures had no significant differences from that of negative controls (the median survival time were 8, 8, and 9 days, respectively). The DCpIL-10 and the DCpIL-10^+^pMHC tolerogenic cultures extended median graft survival time to 12 days (Figure 7(c)). These observations thus highlighted the nonspecific nature of graft rejection suppression when using the pIL-10-transfected DCs and antigen-specific nature of graft rejection suppression when using the pMHC-transfected DCs.

## 4. Discussion

In this study, we investigated two different approaches to suppress transplantation reactions: the use of IL-10-producing DCs and the use of antigen-loaded DCs. IL-10-producing DCs were obtained by electroporation of DCs with a plasmid encoding IL-10 (pIL-10). To generate alloantigen-loaded DCs, we electroporated C57BL/6 DCs with a plasmid DNA construct encoding antigenic determinants from the H2 locus of CBA mice which differ from the homologous antigenic determinants of C57BL/6 mice (pMHC). We assessed the effectiveness of the *in vivo* and *in vitro* allogeneic immune response suppression using cocultures of the transfected DCs and autosplenocytes.

Novel plasmid DNA constructs encoding distinct homologous determinants from the MHC genes were generated using database information thus enabling to avoid the use of donor material for obtaining the alloantigen-loaded DCs. It should be noticed that this approach may be relevant for further clinical applications of tolerogenic cell-based immunotherapy.

In our experiments, the DCs electroporated with the plasmid DNA constructs had more mature phenotypes compared with the nonelectroporated immature DCs (Figure 2). This effect was associated with electroporation itself inducing the maturation of DCs [12]. However, despite the more mature phenotype, the DCs transfected with pIL-10 as well as with pMHC induced FoxP3^+^ Tregs and IL-10^+^CD4^+^ cells in cocultures with autologous splenocytes at similar levels to immature nonelectroporated DCs (Figure 3). These findings correspond to the researches showing that immature DCs [17, 18], IL-10-producing DCs [5, 6, 21–23], and antigen-loaded DCs [8, 17, 18] are able to induce CD4^+^IL-10^+^ cells and FoxP3^+^ Tregs. In our opinion, the level of suppression which corresponds to that of nonelectroporated immature DCs is enough and expected, as the effect of this group, in fact, more closely reflects that of the immature DCs taking part in immunoregulatory processes *in vivo* than other groups. It is also should be taken into account that the DCs transfected with control p5 plasmid failed to induce CD4^+^IL-10^+^ cells and FoxP3^+^ Tregs. This fact confirms the effectiveness of using experimental plasmids, as the p5 plasmid encodes the only antigenic determinants from the H2 locus of CBA mice which do not differ from the homologous antigenic determinants of C57BL/6 mice having more mature phenotype after electroporation and lack of antigen loading and IL-10 secretion.

In MLCs, we observed an ability of the DCs transfected with pIL-10, pMHC, and pIL-10^+^pMHC to suppress the proliferation of autosplenocytes in response to CBA alloantigens *in vitro* (Figure 4). However, only the pIL-10-transfected DCs could suppress the *in vitro* proliferation of autosplenocytes in response to third-party BALB/c alloantigens. This showed that DCs transfected with pMHC (encoding antigenic determinants from the H2 locus of CBA mice which differ from the homologous antigenic determinants of C57BL/6 mice) suppressed *in vitro* immune reaction in an antigen-specific manner.

Acute GVHD induced by C57Bl/6 splenocytes injection to CBF1 mice (F1: CBA×C57Bl/6) was accompanied by splenomegaly, expansion of CD3^+^ and CD8^+^, and reduction of CD19^+^ and CD4^+^ spleen lymphocytes (Supplementary Figure S4), which are known to be acute GVHD signs [24, 25].

In the semiallogeneic GVHD model used, tolerogenic cultures containing pIL-10-transfected DCs reduced GVHD signs 2 weeks after the GVHD induction (Figure 5). Three weeks after the GVHD induction, this effect was less pronounced. Tolerogenic cultures containing pMHC-transfected DCs reduced GVHD signs 3 weeks post-GVHD induction but not after 2 weeks. Tolerogenic cultures containing pIL-10^+^pMHC-transfected DCs were capable to reduce GVHD signs 2 and 3 weeks after the induction.

The analysis of CD45^+^CD4^+^C25^+^ activated cells showed that all used DCs groups failed to decrease the frequency of CD25^+^ “activated” cells (Suppl. Fig. S5). So the generated DCs appeared not to induce clonal T cell anergy, if talk in terms of dividing the anergy state into clonal and adaptive one [26]. It is possible, however, that the CD25^+^ lymphocytes generated in the presence of antigenic stimulation and a high degree of coinhibition may be in the adaptive anergy state [26] which does not prevent the lymphocyte proliferation but limits its effector function [26]. Further investigation is needed to clarify the exact type of lymphocytes anergy obtained. Interestingly, increased FoxP3^+^ Treg frequencies were observed only in the DCpMHC and the DCpIL-10^+^pMHC groups 3 weeks post-GVHD induction despite the fact that DCs transfected with IL-10 were capable of Treg induction in MLCs. According to the previous findings, *in vivo* Treg generation by IL-10-producing DCs requires allogeneic [21] or antigen-loaded [27] DCs. By contrast, Tregs can be generated *in vitro* with syngeneic non-antigen-loaded IL-10 producing DCs [22]. This difference linked with that IL-10-induced FoxP3 expression is transient since the loss of IL-10 signaling results in loss of FoxP3 expression [28], and when transient FoxP3 expression is excluded from analysis, IL-10 does not influence the level of FoxP3 expression [29]. Stable FoxP3 expression is known to require additional stimuli such as T cell receptor signaling at the proper intensity, costimulatory molecule interactions, PD-1/PD-L1 interactions, and indoleamine 2,3-dioxygenase activity [30]. Thus, in MLC were observed Tregs with transient FoxP3 expression induced by IL-10, which may be lost after reduction of IL-10 signaling *in vivo*.

In fully allogeneic GVHD models with BALB/c mice, only DCs transfected with pIL-10 could reduce GVHD signs 3 weeks after induction (Figure 6). Three weeks post-GVHD induction, we did not observe higher Treg frequencies in the host BALB/c spleens of the DCpMHC and DCpIL-10^+^pMHC groups unlike in CBA hosts.

Therefore, the results of our GVHD experiments suggest that suppression of GVHD by DCs transfected with pMHC (encoding antigenic determinants from the H2 locus of CBA mice, which differ from the homologous antigenic determinants of C57BL/6 mice) was antigen-specific and followed by expansion of FoxP3^+^ Treg cells. Suppression of GVHD by DCs transfected with pIL-10 was antigen-independent.

In skin graft transplantation experiments, all experimental treatment options (DCpIL-10, DCpMHC, and DCpIL-10^+^MHC) extended CBA skin graft survival time (Figure 7). The extension of skin graft survival using these treatment options was comparable to the extension of graft survival time using rapamycin or cyclosporin [31] and immature DCs pulsed with allo-MHC peptides [12] but shorter than in some cases of using immature allo-DCs [9]. Thus, although the provided approach eliminates the necessity for donor material use, it needs further improvement and optimization to achieve longer graft survival time.

Only DCpIL-10 and DCpIL-10^+^pMHC were capable of extending BALB/c skin graft survival, highlighting the antigen-specific mechanism of DCpMHC action.

## 5. Conclusions

In summary, our data suggest that pIL-10-transfected DCs and pMHC-transfected DCs suppress transplantation reactions using different nonspecific and antigen-specific mechanisms, respectively. Since immune tolerance is defined as antigen-specific suppression of immune reactions [32] and the known mechanisms of immune tolerance are lymphocyte anergy [26] and Treg induction [33], that allows us to state that the *in vitro* hyporeactivity to CBA antigens and the *in vivo* suppression of graft rejection and GVHD to CBA antigens accompanied by Tregs generation induced by DCs transfected with MHC antigenic determinants refers to manifestations of immune tolerance. Despite the pIL-10-transfected DCs were able to suppress transplantation reactions, their action was nonspecific and did not match immune tolerance properties.

Authors suppose that the induction of antigen-specific immune tolerance to transplant antigens may decrease the need for nonspecific immunosuppressive therapy accompanied by numerous side effects. In the present work, we offered the approach for the induction of antigen-specific immune tolerance to transplant antigens with the use of DCs transfected with preliminarily designed DNA constructs encoding MHC antigenic determinants differ between donor and recipient which eliminates the necessity of donor material use.

## Data Availability

The datasets generated during the current study are available from the corresponding author on request.

## Abbreviations

DCs: Dendritic cells
GVHD: Graft-versus-host disease
IL: Interleukin
MHC: Major histocompatibility complex
MLC: Mixed lymphocyte culture
OD: Optical density
PBS: Phosphate-buffered saline
rmGM-CSF: Recombinant mouse granulocyte-macrophage colony-stimulating factor
tol-DCs: Tolerogenic dendritic cells
Tregs: Regulatory T lymphocytes.

## Supplementary Materials

Supplementary 1 Antigenic determinants of H2 locus of CBA mice encoded by pMHC plasmids and their differences from homologous fragments of C57Bl/6 mice.

Supplementary 2 (A) DC gating strategy. (B) Representative histograms of DCs phenotype staining.

Supplementary 3 Flow cytometry scatter plots and gating strategies. (a) Gating strategy for the identification of CD4^+^C25^+^FoxP3^+^ Tregs. (b) Relative numbers of Tregs in cultures of splenocytes stimulated with different groups of transfected DCs. (c) Gating strategy for the identification of CD4^+^IL-10^+^ cells. (d) Relative numbers of CD4^+^IL-10^+^ cells in splenocyte cultures stimulated with different groups of transfected DCs.

Supplementary 4 Acute GVHD signs at different time points (7, 14, and 21 days) after injection of C57Bl/6 splenocytes to CBF1 (F1: C57Bl/6 × CBA). Injection of saline (circles), 5 × 10^7^ splenocytes (squares), 1 × 10^8^ splenocytes (triangles), and 1.5 × 10^8^ splenocytes (inverted triangles). (a) Spleen weight. (b–e) Relative numbers of spleen CD3^+^, CD19^+^, CD8^+^, and CD4^+^ lymphocytes, respectively.

Supplementary 5 The frequency of CD45^+^CD4^+^CD25^+^ cells in CBF1 mouse splenocytes 2 weeks (A) and 3 weeks (B) after GVHD induction.
