# Supplementary material for: Dendritic Cells Transfected with MHC Antigenic Determinants of CBA Mice Induce Antigen-Specific Tolerance in C57Bl/6 Mice
Source: J Immunol Res. 2020 Sep 4;2020:9686143. doi: 10.1155/2020/9686143 (PMC7487104; doi:10.1155/2020/9686143)
Supplement: Supplementary 1 — Antigenic determinants of H2 locus of CBA mice encoded by pMHC plasmids and their differences from homologous fragments of C57Bl/6 mice. [file 9686143.f1.docx]

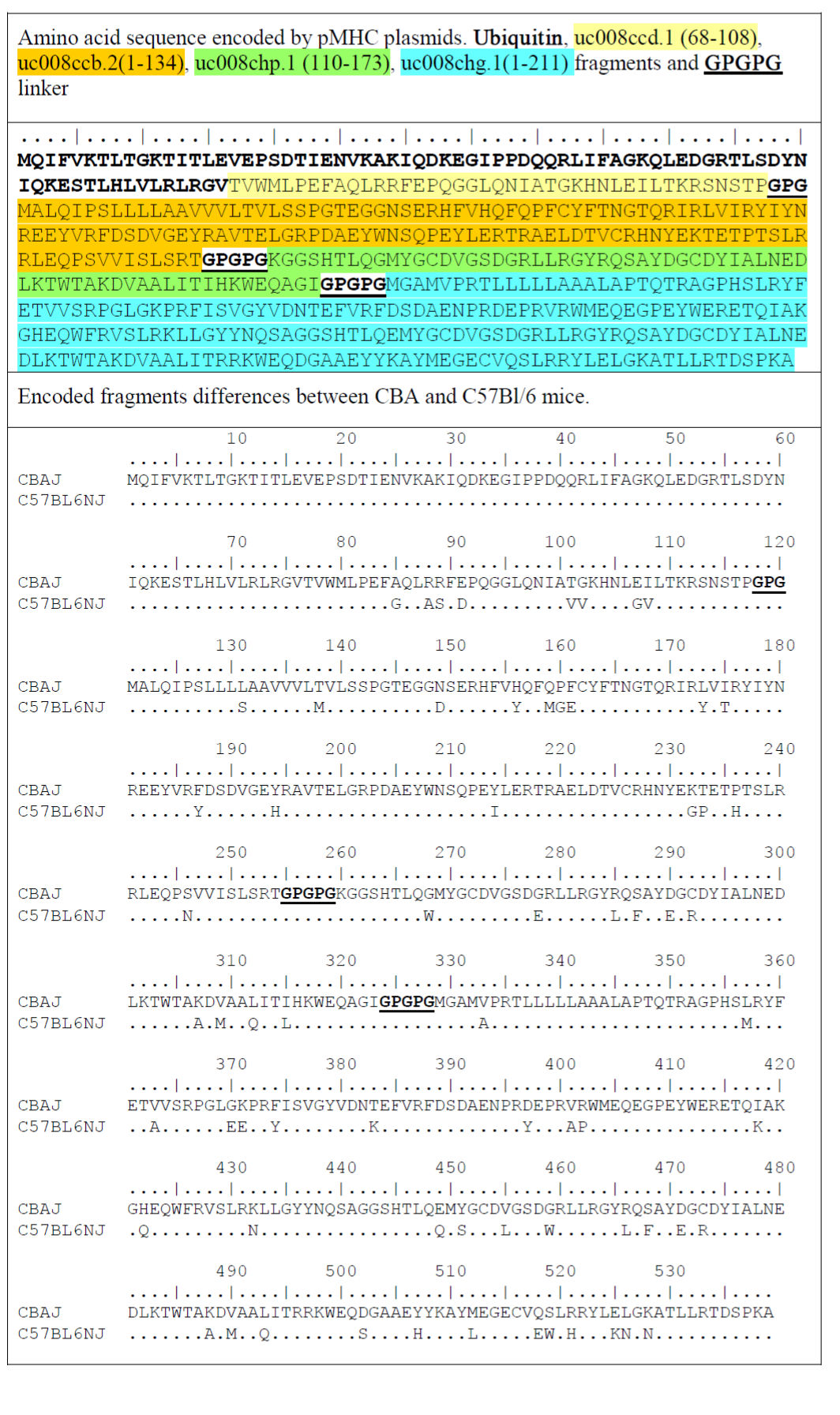
**Supplementary Figure S1.** Antigenic determinants of H2 locus of CBA mice encoded by pMHC plasmids and their differences from homologous fragments of C57Bl/6 mice.
