# Supplementary material for: Dendritic Cells Transfected with MHC Antigenic Determinants of CBA Mice Induce Antigen-Specific Tolerance in C57Bl/6 Mice
Source: J Immunol Res. 2020 Sep 4;2020:9686143. doi: 10.1155/2020/9686143 (PMC7487104; doi:10.1155/2020/9686143)
Supplement: Supplementary 2 — (A) DC gating strategy. (B) Representative histograms of DCs phenotype staining. [file 9686143.f2.docx]

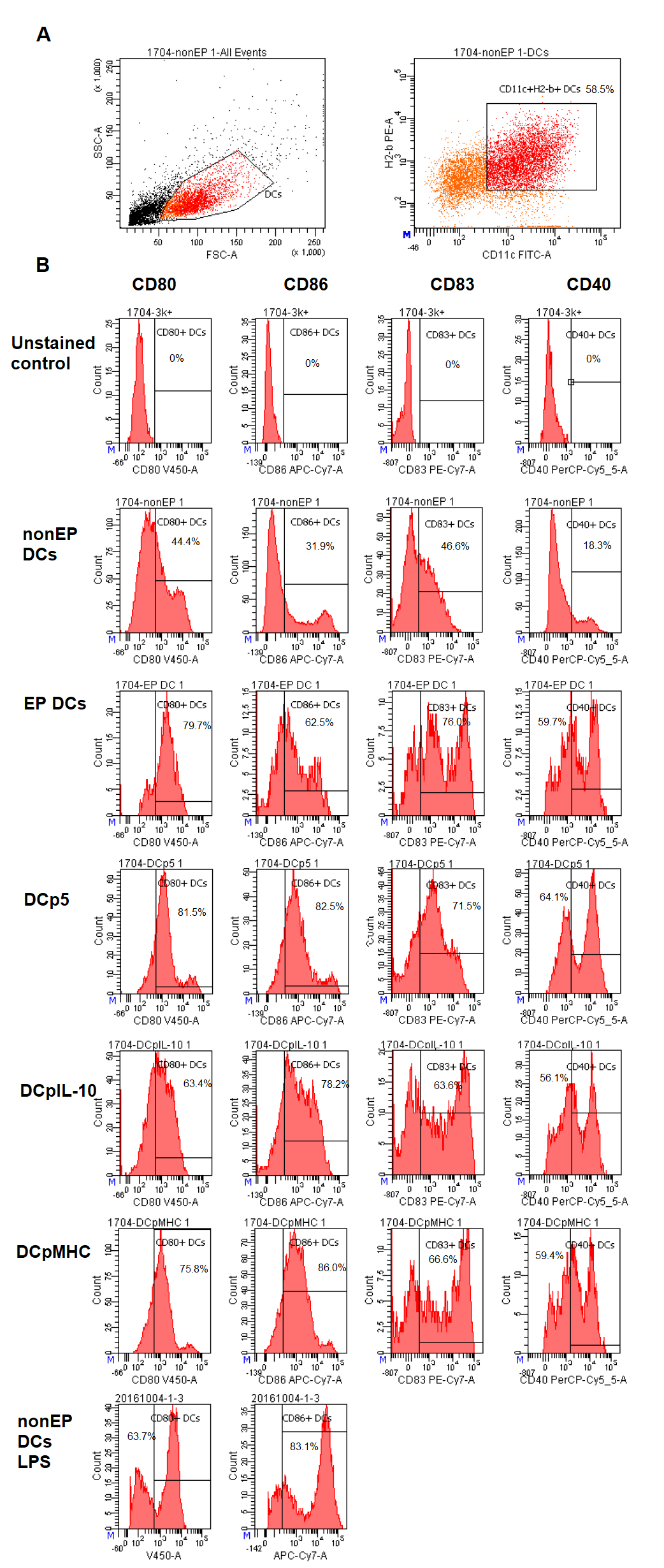


**Supplementary figure S2**. **A** – DCs gating strategy. **B** – representative histograms of DCs phenotype staining.
