# Supplementary material for: Dendritic Cells Transfected with MHC Antigenic Determinants of CBA Mice Induce Antigen-Specific Tolerance in C57Bl/6 Mice
Source: J Immunol Res. 2020 Sep 4;2020:9686143. doi: 10.1155/2020/9686143 (PMC7487104; doi:10.1155/2020/9686143)
Supplement: Supplementary 3 — Flow cytometry scatter plots and gating strategies. (a) Gating strategy for the identification of CD4+C25+FoxP3+ Tregs. (b) Relative numbers of Tregs in cultures of splenocytes stimulated with different groups of transfected DCs. (c) Gating strategy for the identification of CD4+IL-10+ cells. (d) Relative numbers of CD4+IL-10+ cells in splenocyte cultures stimulated with different groups of transfected DCs. [file 9686143.f3.docx]

**Supplementary figure S3.** Flow cytometry scatter plots and gating strategies. **(a)** — Gating strategy for the identification of CD4^+^C25^+^FoxP3^+^ Tregs. **(b)** — Relative numbers of Tregs in cultures of splenocytes stimulated with different groups of transfected DCs. **(c)** — Gating strategy for the identification of CD4^+^IL-10^+^ cells. **(d)** — Relative numbers of CD4^+^IL-10^+^ cells in splenocyte cultures stimulated with different groups of transfected DCs.

**
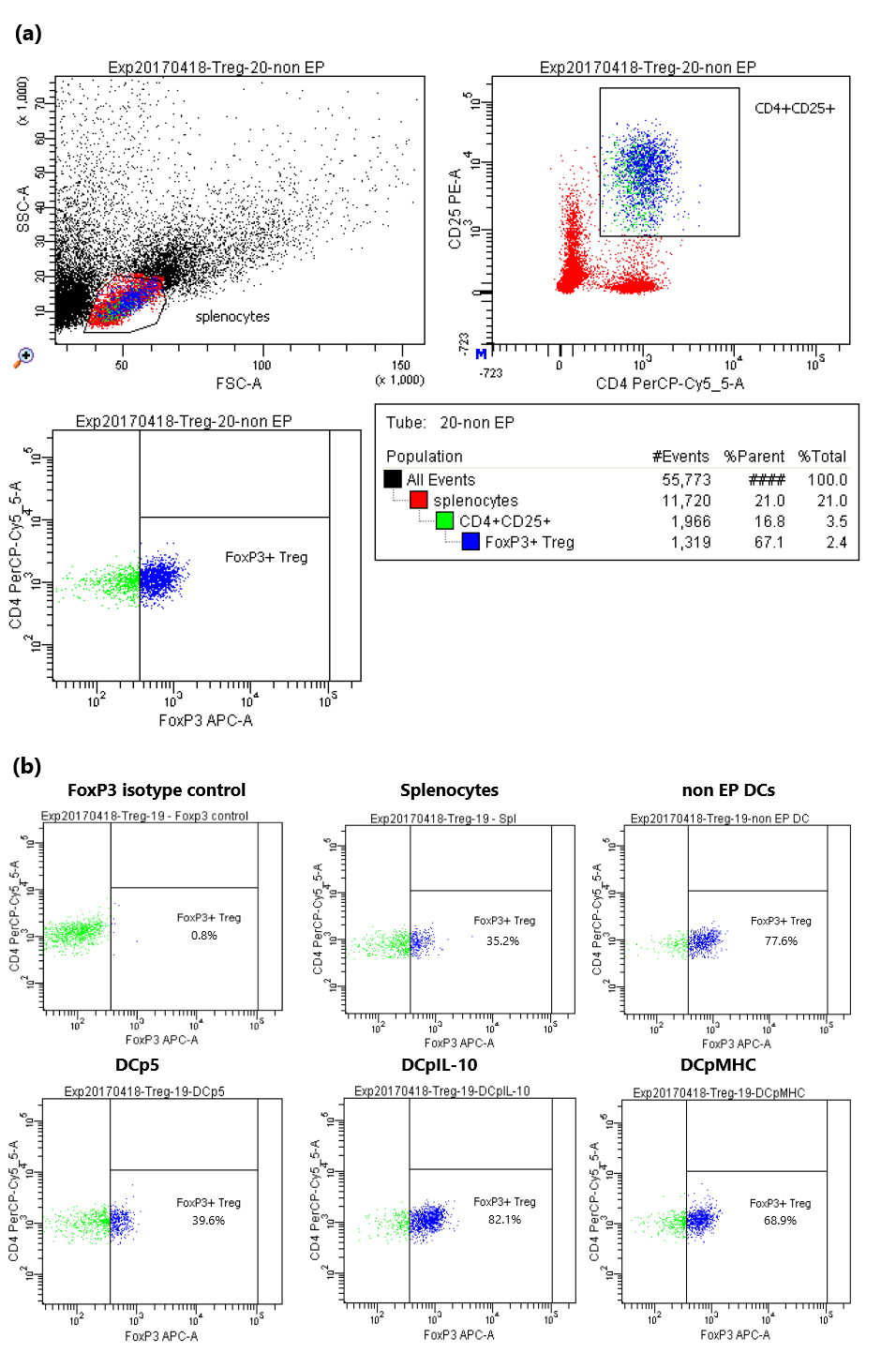
**

**
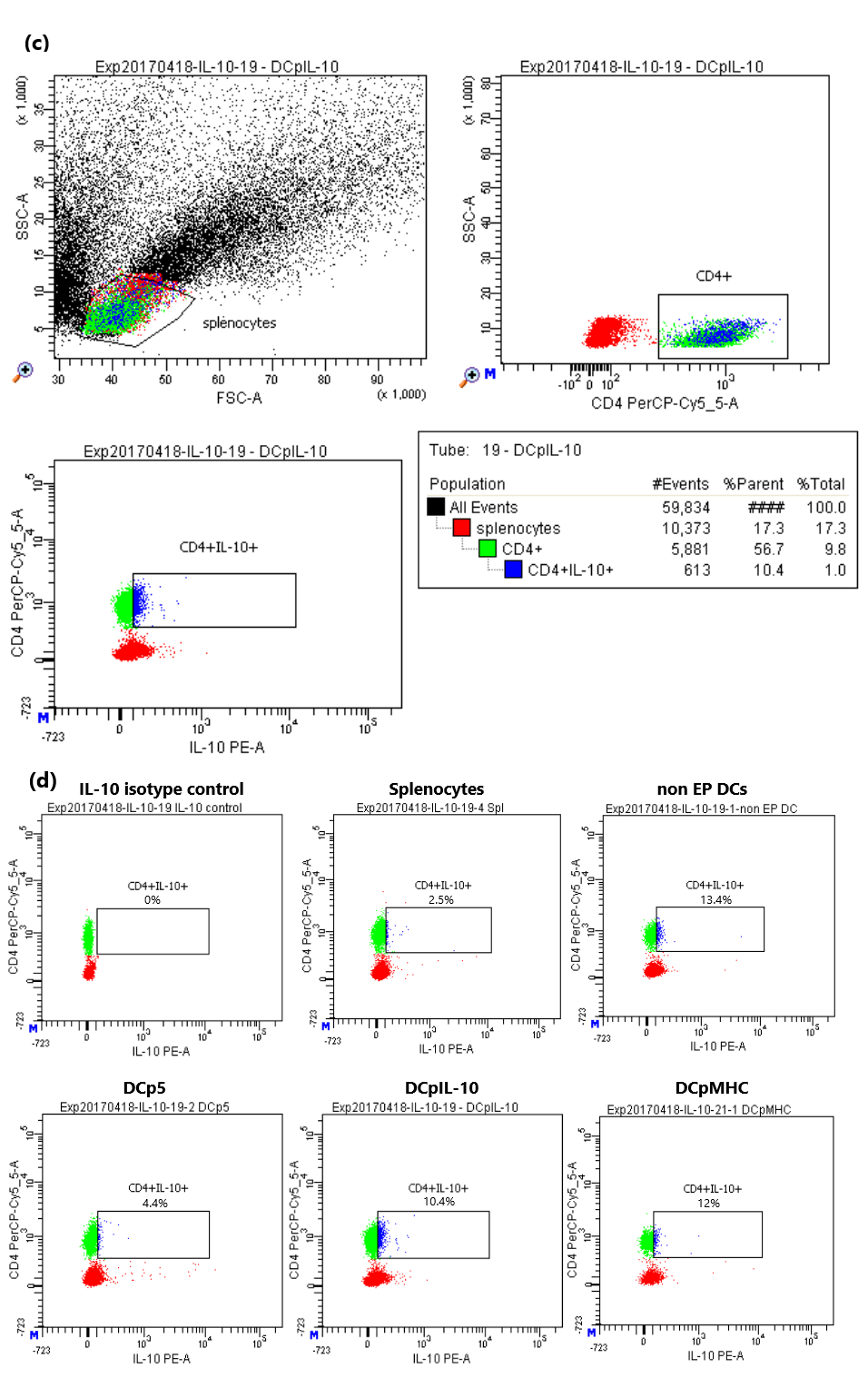
**
