# Supplementary material for: Dendritic Cells Transfected with MHC Antigenic Determinants of CBA Mice Induce Antigen-Specific Tolerance in C57Bl/6 Mice
Source: J Immunol Res. 2020 Sep 4;2020:9686143. doi: 10.1155/2020/9686143 (PMC7487104; doi:10.1155/2020/9686143)
Supplement: Supplementary 4 — Acute GVHD signs at different time points (7, 14, and 21 days) after injection of C57Bl/6 splenocytes to CBF1 (F1: C57Bl/6 × CBA). Injection of saline (circles), 5 × 10^7^ splenocytes (squares), 1 × 10^8^ splenocytes (triangles), and 1.5 × 10^8^ splenocytes (inverted triangles). (a) Spleen weight. (b–e) Relative numbers of spleen CD3+, CD19+, CD8+, and CD4+ lymphocytes, respectively. [file 9686143.f4.docx]

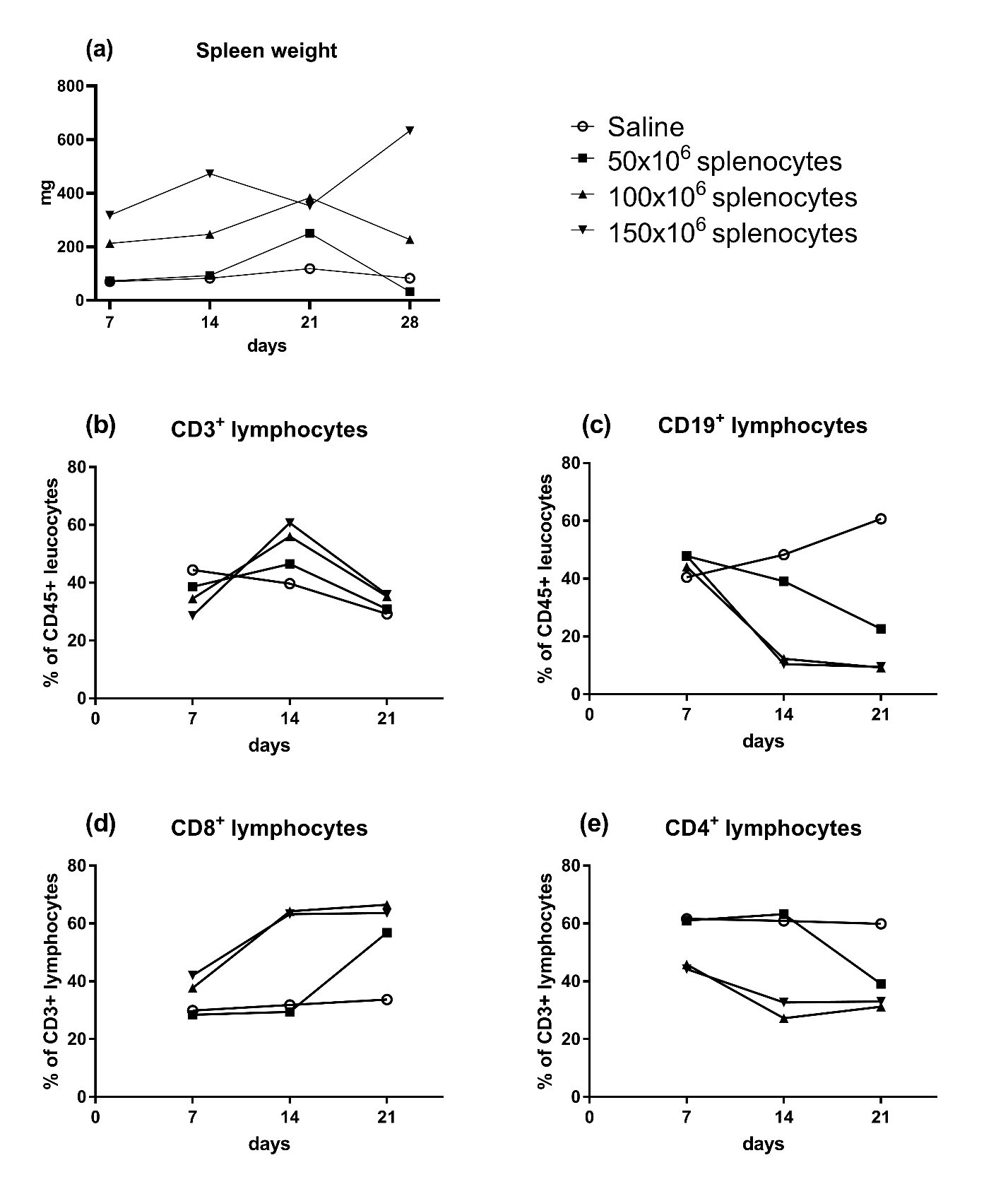
**Supplementary Figure S4.** Acute GVHD signs at different time points (7, 14, 21 days) after injection of C57Bl/6 splenocytes to CBF1 (F1: C57Bl/6 × CBA). Injection of saline (circles), 5×10^7^ splenocytes (squares), 1×10^8^ splenocytes (triangles), 1,5×10^8^ splenocytes (inverted triangles). **(a)** — spleen weight. **(b), (c), (d), (e)** — relative numbers of spleen CD3^+^, CD19^+^, CD8^+^, CD4^+^ lymphocytes, respectively.
