# Supplementary material for: Dendritic Cells Transfected with MHC Antigenic Determinants of CBA Mice Induce Antigen-Specific Tolerance in C57Bl/6 Mice
Source: J Immunol Res. 2020 Sep 4;2020:9686143. doi: 10.1155/2020/9686143 (PMC7487104; doi:10.1155/2020/9686143)

**Supplementary figure S5.** The frequency of CD45^+^CD4^+^CD25^+^ cells in CBF1 mice splenocytes 2 weeks(A) and 3 weeks(B) after GVHD induction.
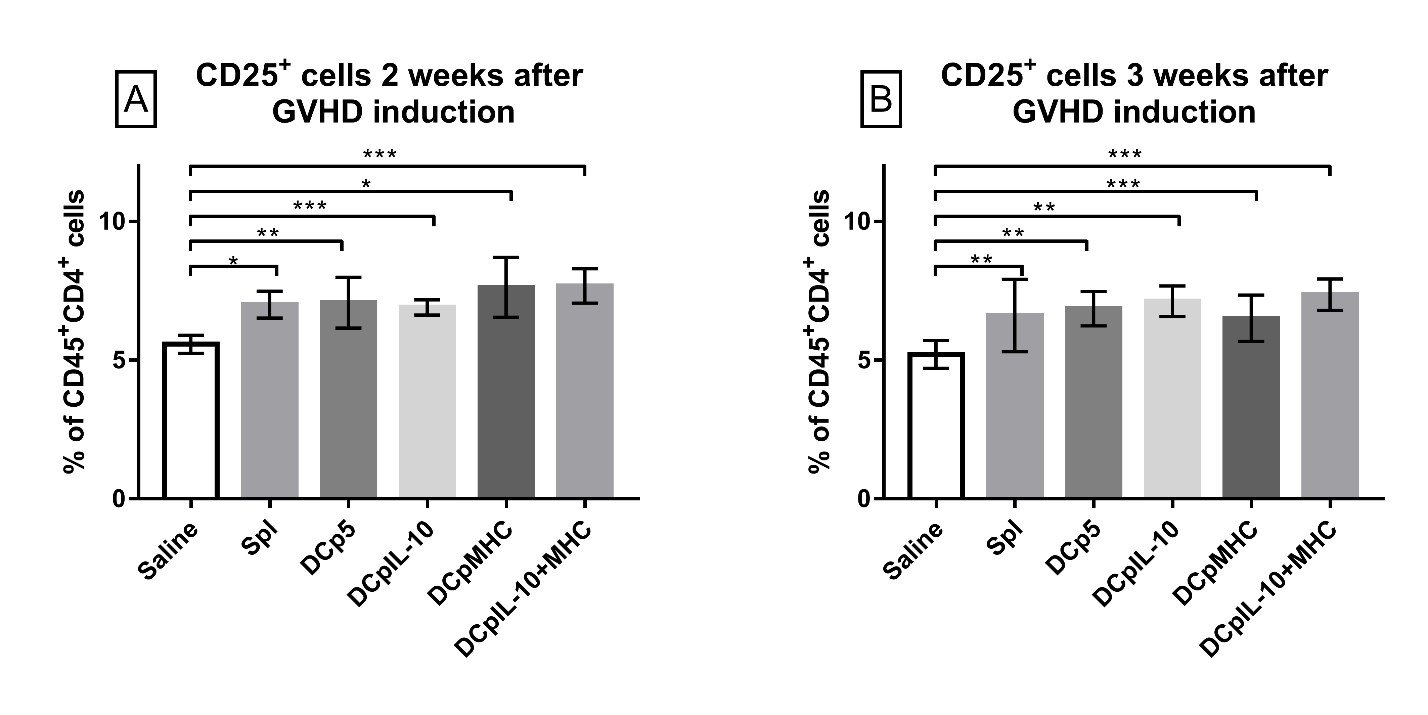

Supplement: Supplementary 5 — The frequency of CD45+CD4+CD25+ cells in CBF1 mouse splenocytes 2 weeks (A) and 3 weeks (B) after GVHD induction. [file 9686143.f5.docx]
